# Effect of pessary cleaning and optimal time interval for follow-up: a prospective cohort study

**DOI:** 10.1007/s00192-019-04200-8

**Published:** 2020-01-06

**Authors:** Susanne D. Thys, Robert A. Hakvoort, Joyce Asseler, Alfredo L. Milani, Astrid Vollebregt, Jan Paul Roovers

**Affiliations:** 1 Amsterdam University Medical Center, Meibergdreef 9, 1105 AZ Amsterdam, The Netherlands; 2 Martini Hospital, Groningen, The Netherlands; 3 Spaarne Gasthuis, Hoofddorp, The Netherlands; 4 Reinier de Graaf Groep, Delft, The Netherlands

**Keywords:** Pessary, Cleaning, Follow-up interval, Self-management, Pelvic organ prolapse

## Abstract

**Introduction and hypothesis:**

The objective of this study was to determine the efficacy of routine follow-up visits for pessary cleaning, the effect of extended time intervals between visits and the proportion of patients being able to self-manage their pessary for pelvic organ prolapse (POP).

**Methods:**

We conducted a prospective cohort study in patients with a stage ≥II POP without previous POP surgery. All patients received a pessary as primary treatment. Our main outcome measure was a difference ≥2 in median visual analogue scale (VAS) scores (for pain, discharge, irritation) 1 week before and 1 week after cleaning. Measurements were performed after 3- and 9-month cleaning intervals. For the evaluation of the effect of cleaning, 132 patients (3 months’ follow-up) and 87 patients (12 months’ follow-up) were available for analysis. For the evaluation of the effect of the lengthening interval, 123 patients were available.

**Results:**

Self-management was performed in 45.2% of patients at 1 year. In 93.1% of patients, no differences were observed in pre-and post-cleaning VAS scores (effect of cleaning) on vaginal pain. Nor was there a difference in discharge (72.4%) or irritation 85.1% (*p* = 0.00). No differences were observed in pre-cleaning VAS scores for vaginal pain, discharge and irritation when the interval was lengthened from 3 to 9 months. No serious adverse events occurred.

**Conclusions:**

There is no proven benefit of regular follow-up visits to clean a pessary. Also, the length of the cleaning interval does not seem to matter.

## Introduction

Pelvic organ prolapse (POP) is a common condition in adult women [1, 2]. The prevalence of symptomatic POP in the general population is 11.4% (4–12.2%) [2]. Apart from pelvic floor muscle training, there are two other management options for POP; the use of a vaginal pessary and pelvic reconstructive surgery. As a non-surgical treatment option, vaginal pessaries are often used as first-line treatment in patients who do not want surgery, patients who are at a higher risk of surgical complications or in patients with recurrent POP [1]. Reasons to start pessary therapy are reducing POP-related symptoms, preventing progression of prolapse and avoiding surgery [1, 3].

Compared with surgery, advantages of pessary use are the low rate of complications and the immediate effectiveness [4–6]. However, disadvantages also exist. The most frequently described side effects of pessaries are vaginal discharge (25–33%), vaginal irritation/pain (2–33%) and vaginal blood loss (6–46%) [4, 5, 7–10]. Rarely, cases of more serious adverse events of pessary use are described, such as fistula, hydronephrosis, urosepsis, incarceration and carcinoma [1, 4, 5, 11]. However, it must be noted that these serious adverse events are often due to longstanding neglect of pessaries [4, 5, 12].

Whether a pessary will be chosen as first-line treatment depends on expectations about the effect, the likelihood of occurrence of side effects and patients’ preferences [13, 14] and existing guidelines advising to try the least invasive option initially [6]. Overall, in the Netherlands, this has resulted in high acceptance, with up to 98% of clinicians reporting use of pessaries in the management of POP and even 77% of gynaecologists reporting use of pessaries as first-line treatment [15, 16].

With this widespread use of pessaries, it becomes even more relevant to optimise management protocols. In comparison with surgery, short-term costs of pessaries are lower. However, long-term costs are highly dependent on the need and frequency of follow-up visits. These follow-up visits involve removal and cleaning of the pessary, inspection of the vaginal wall and afterwards replacing the pessary. These visits can be a burden to patients. Equally important, we do not know whether these visits are effective in reducing pessary related side effects. Further, intervals between these follow-up visits vary widely and studies about the ideal length of such intervals are lacking [1, 7, 11, 16, 17]. Nor is it known what proportion of patients is willing and able to learn how to clean and replace the pessary themselves.

To optimize treatment protocols and optimize the number of follow-up visits, we performed a prospective cohort study to evaluate whether or not cleaning a pessary has any effect on pessary-related symptoms such as vaginal discharge, vaginal irritation and pain. In addition, we evaluated if pessary-related side effects prior to the cleaning differ when the interval is significantly lengthened from 3 to 9 months.

## Materials and methods

### Study design and setting

A prospective cohort study of women with symptomatic pelvic organ prolapse (POP) and a POP-Q [18] stage 2 or higher was performed between January 2014 and December 2017 to determine the percentage of continued users after 12 months. In this cohort, several secondary outcomes were measured: disease-specific quality of life, sexual function after 1 year, factors related to discontinuation of pessary use, the effect of cleaning a pessary and pessary-related complaints when intervals between visits were increased from 3 to 9 months’ follow-up. In this article we present the data concerning:

- The effectiveness of cleaning a pessary regarding the complaints of vaginal pain, discharge and irritation.
- The comparison between a 3-month cleaning interval with a 9-month interval concerning the complaints of vaginal pain, discharge and irritation.
- The study was conducted in three teaching hospitals. Study approval was obtained from the Institutional Review Board (IRB).

### Participants

All patients who visited the outpatient clinic with primary, symptomatic POP (feeling or seeing a bulge) and a prolapse stage 2 or higher according to the POP-Q classification were eligible to participate and consequently given information about the study. Patients who completed the 3-month follow-up visit with the intention of continuing pessary use were included in this study. Patients with previous pelvic reconstructive surgery for POP or stress urinary incontinence (SUI) were not included.

### Study procedure and follow-up

When treatment for prolapse was preferred, all patients initially received a silicone ring pessary (with or without support). Choice regarding the size of pessary was left to the discretion of the gynaecologist. All patients were offered education concerning self-management, which included practical explanation about removal, cleaning and replacement of the pessary. In the case of vaginal bleeding or excessive vaginal discharge, patients were instructed to visit their gynaecologist before the intended first follow-up visit.

The following baseline characteristics were noted in a case record form (CRF): age, parity, POP-Q stage, presence of vaginal atrophy and any prescription of local oestrogen treatment, complaints of concomitant SUI and sexual activity.

The first standard follow-up visit after insertion of the pessary was 3 months after initial placement of the pessary. In this visit, the pessary was removed and cleaned. The vaginal wall was inspected for lesions. When there were no signs of complications and the patient wished to continue pessary treatment, the pessary was replaced. To measure the effect of pessary cleaning, patients were asked to rate three known pessary-related side effects using a 1–10 visual analogue scale (VAS): vaginal pain, discharge and irritation [4, 7]. On this scale, the value 1 reflects no complaints of this side-effect and 10 reflects very severe complaints of this side-effect. This measurement was performed 1 week prior to the follow-up visit and 1 week after this follow-up visit. As a wide range of (clinically relevant) differences in VAS scores was reported and, taking errors of this measurement tool into account, we considered a difference in VAS scores of ≥2 to be a clinically significant difference [19]. To answer the second question, whether or not pessary-related side effects differ when intervals increase from 3 to 9 months, only measurements before follow-up visits were needed.

In addition, patients received a self-developed questionnaire about whether or not they had performed self-management. If they had performed self-management, patients were asked about the frequency of self-management (daily, weekly, monthly). The second follow-up visit was 12 months after insertion of the pessary (9-month interval). Before, during and after this visit the same procedure was repeated as described above.

A sub-analysis was performed on the effect of cleaning and lengthening of the cleaning interval between patients with and without self-management.

### Statistical analysis

All data were entered and analysed in a Statistical Package for the Social Sciences (SPSS Statistics for Windows, version 24.0; IBM, Armonk, NY, USA). Descriptive analyses were performed as appropriate for the respective variable. First, the normality of the variable was determined. Means with standard deviations were given for continuous, normally distributed data, medians with quartiles for not normally distributed continuous data and frequencies with percentages for categorical data. Comparisons were made using Wilcoxon signed rank test for continuous not normally distributed paired data, Mann–Whitney *U* test for continuous not normally distributed unpaired data and Chi-squared testing for categorical data. A *p* value of less than 0.05 was considered a threshold for statistical significance.

## Results

**Fig. 1:**
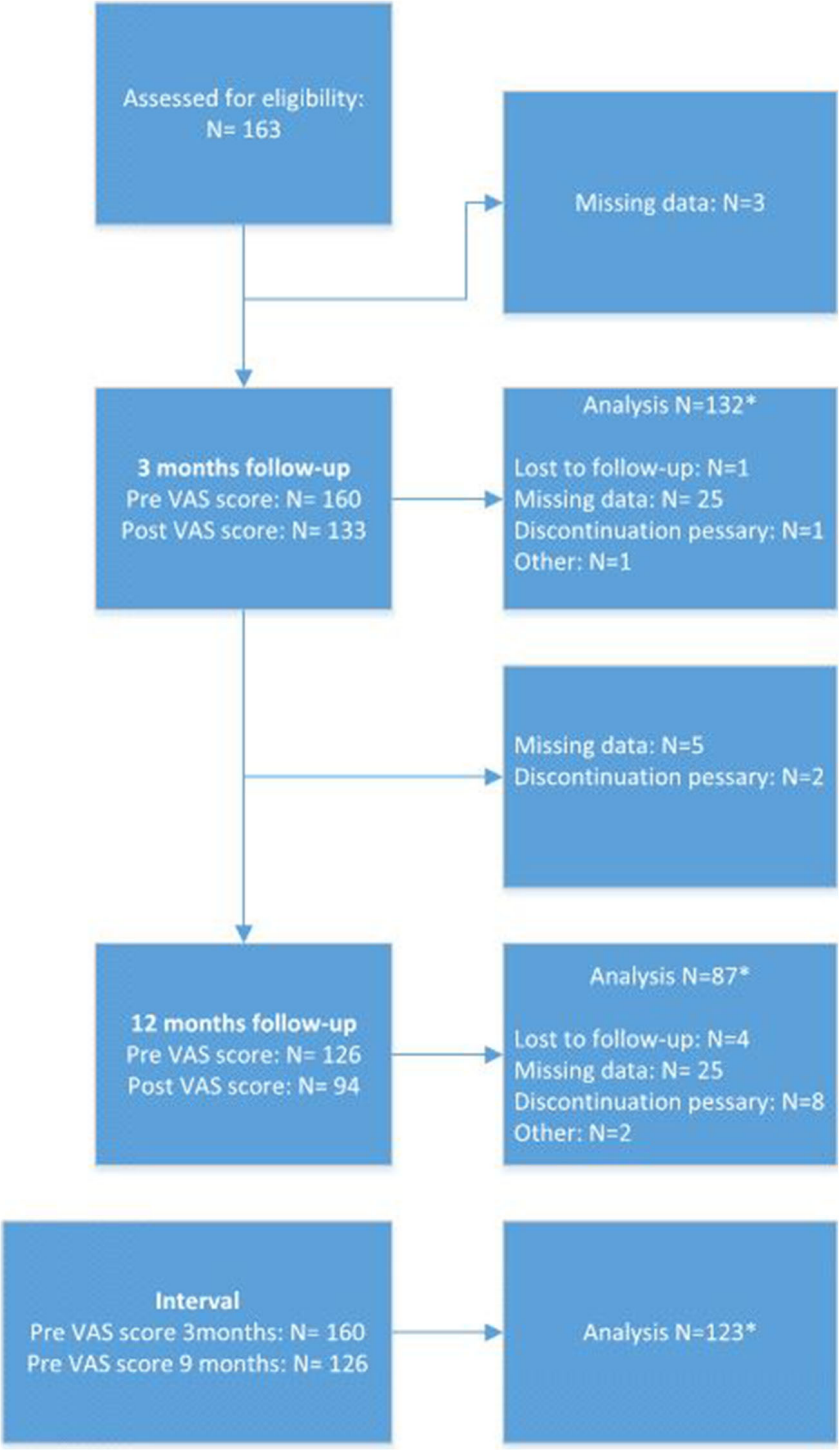
Flowchart. *Measurements in the same patient. All patients with missing data have continued pessary use at 12 months’ follow-up

Between January 2014 and December 2016, there were 365 patients eligible for the prospective cohort study. A total of 163 pessary users continued the use of a pessary after 3 months’ follow-up and were included. Of these patients, there were 132-paired measurements before and after cleaning at 3 months’ follow-up and 87 paired measurements before and after cleaning at 12 months’ follow-up. With these measurements, it could be investigated whether cleaning a pessary had any effect on pessary-related complaints (i.e. vaginal pain, discharge and irritation). Of 123 patients, there was a pre-VAS score measurement available at both the 3- and the 12-month follow-up (Fig. 1).

Figure 1 shows the patients who were lost to follow-up and the missing data. In total, 5 patients (3%) were lost to follow-up. One of them died (not pessary-related) and the other four did not return to any follow-up visit, nor did they respond to requests to make an appointment. Eleven (6.7%) patients discontinued pessary use, of which 7 (4.3%) underwent pelvic reconstructive surgery. Four patients refused further pessary treatment and operative treatment for POP for several reasons, e.g. discharge complaints and no wish for surgery. Further, in one patient treatment was temporarily stopped because of a lesion of the vaginal epithelium due to pessary use, one patient changed to a cube pessary and the other patient only used a pessary while exercising. All patients in the “missing data” group continued pessary treatment (according to the patient record files).

Table 1 shows the baseline characteristics of the study population (and divides into patients with and without self-management). The mean age was 65 years (75–72), of which the majority were post-menopausal (88.9%). Median parity was 2 (range 2–3). One hundred and one (64.3%) patients had a POP-Q classification stage 2, 55 (35.0%) stage 3 and 1 (0.7%) had stage 4 prolapse. Vaginal atrophy was present in 63 patients (44.1%) of whom 51 (81%) were treated with vaginal oestrogen. Concomitant SUI was reported in 32 patients (21.2%). Sexual activity was present in 50.3% of our study population, 94% of them with vaginal penetration.

**Table 1:** Characteristics in the total study population and in women with or without self-management

	Total (*N* = 163)		No self-management (*N* = 70)		Self-management (*N* = 74)		*p* value
Age	65	57–72	67	60–74	63	56–70	0.04*
Parity	2	2–3	2	2–3	2	2–3	0.52*
Menopausal state							
Premenopausal	11	6.8	2	3.0	7	9.7	0.39**
Perimenopausal	7	4.3	4	6,00	3	4.2	
Postmenopausal	143	88.9	61	91.0	62	86.1	
POP-Q							
Stage 2	101	64.3	47	72.3	40	54.8	0.08**
Stage 3	55	35.0	18	27.7	32	43.8	
Stage 4	1	0.7	0	0	1	1.4	
Vaginal atrophy							
Yes	63	44.1	17	28.8	42	60.0	0.00**
Vaginal HT	51	35.7	12	20.3	37	52.9	
No	80	55.9	42	71.2	28	40.0	
SUI							
Yes	32	21.1	15	25.4	14	19.2	0.01**
No	119	78.8	44	74.6	59	80.8	
Sexually active							
Yes	80	50.3	26	38.2	44	61.1	0.03**
Penetration	75	47.2	25	36.8	41	56.9	
No	79	49.7	42	61.8	28	38.9	

Data are median (quartiles) or numbers (percentage)

*SUI* stress urinary incontinence

*Mann–Whitney *U* test, **Chi-squared test

Patients who performed self-management were younger (median 63 vs 67 years respectively *p* = 0.04), were more frequently diagnosed with vaginal atrophy (60 versus 28.8% respectively, *p* = 0.00), reported less concomitant SUI (80.8 versus 74.6% respectively, *p* = 0.01) and were more often sexually active (including vaginal penetration; 61.1 vs 38.2% respectively; *p* = 0.03).

The effect of cleaning is shown in Tables 2 and 3. Overall, no significant differences were found in pre- and post-cleaning VAS scores (difference of ≥2 points on VAS scores) on vaginal pain, discharge and irritation at the 3- and 12-month follow-up visits (*p* = 0.00 on all vaginal symptoms, in favour of the “no difference” groups). Nevertheless, a number of patients (up to 23% after a cleaning interval of 9 months) tended to have some improvement in vaginal discharge complaints after cleaning. The sub-analysis in patients with self-management and without self-management showed that there were no differences between the two groups (Table 3).

**Table 2:** Pre- and post-cleaning VAS scores on vaginal symptoms

	3 months (*N* = 132)					12 months (*N* = 87)				
	Pre-VAS		Post-VAS		*p* value*	Pre-VAS		Post-VAS		*p* value*
Vaginal pain	1.00	(1–1)	1.00	(1–1)	0.52	1.00	(1–1)	1.00	(1–1)	0.67
Vaginal discharge	3.00	(1–5)	2.00	(1–3)	0.17	3.00	(1–5)	3.00	(1–4)	0.00
Vaginal irritation	1.00	(1–2)	1.00	(1–2)	0.07	1.00	(1–2)	1.00	(1–2)	0.00

Scores are median (25–75 quartiles)

*Wilcoxon signed rank test

**Table 3:** Difference between pre- and post-VAS scores

	3 months (*N*=132)		*p* value*	12 months (*N*=132)		*p* value*	3 months no self-management		*p* value*	3 months self-management		*p* value*	*p* value 2**	9 months no self-management		*p* value*	9 months self-management		*p* value*	*p* value 2**
Vaginal pain																				
Improvement	3	2.3		2	2.3		0	0.0		2	3.1			1	2.6		1	2.2		
Deterioration	3	2.3		4	4.6		2	3.8		0	0.0			2	5.3		2	4.3		
No difference	126	95.4	0.00	81	93.1	0.00	51	96.2	0.00	62	96.9	0.00	0.82	35	92.1	0.00	43	93.5	0.00	0.80
Vaginal discharge																				
Improvement	21	15.9		20	23.0		8	15.1		12	18.8			9	23.7		10	21.8		
Deterioration	15	11.4		4	4.6		7	13.2		7	10.9			1	2.6		3	6.5		
No difference	96	72.7	0.00	63	72.4	0.00	38	71.7	0.00	45	70.3	0.00	0.95	28	73.7	0.00	33	71.7	0.00	0.77
Vaginal irritation																				
Improvement	10	7.6		11	12.6		4	7.5		6	9.4			6	15.8		5	10.9		
Deterioration	4	3.0		2	2.3		3	5.7		1	1.6			0	0.0		1	2.1		
No difference	118	89.4	0.00	74	85.1	0.00	46	86.8	0.00	57	89.0	0.00	0.66	32	84.2	0.00	40	87.0	0.00	0.76

Difference in VAS score (pre-VAS − post-VAS): improvement ≤−2, deterioration ≥2, no difference ≤−1 and ≥1

Data are numbers (percentage)

*Chi-squared test

**Self-management versus no self-management: Mann–Whitney *U* test

No significant differences were observed in vaginal pain (median pre VAS scores 1, *p* = 0.25), discharge (median pre VAS scores 3, *p* = 0.23) and irritation (median pre VAS scores 1, *p* = 0.70) when the interval of cleaning was increased from 3 months and 9 months respectively.

Self-management was performed in 45.2% of included patients compared with 42.9% who did not at 12 months’ follow-up. A remaining 8.2% did not perform self-management at 3 months’ follow-up, but no information about these patients was available at 12 months’ follow-up. In 3.7% of patients, there was no information about self-management at all. Frequencies of performing self-management showed that 5.4% of patients performed this daily, 31.1% weekly, 40.5% monthly and 6.8% yearly. Another 16.2% performed self-management only if they thought it was necessary, of which 8 (10.2%) occasionally went to the general practitioner for help.

No serious adverse events due to pessary use were recorded in our study group after 12 months’ follow-up.

## Discussion

### Main findings

Cleaning a pessary, either by a physician or by the patient herself, does not improve pessary-related side effects. An interval of 6 months between cleaning visits did not result in an increase in pessary-related side effects.

### Strengths and limitations

Strengths of this study are the prospective design (with pre- and post-cleaning measurements) and the relatively large group of patients.

Another strength is that in this study we take a closer look at pessary management and routine follow-up visits. Although there is a wide range of opinions regarding (optimal) pessary management, up to now evidence is still scarce.

Some limitations of our study should be considered.

First, we have to acknowledge that our overall population was a subgroup of successful pessary users, without complications, e.g. lesions of the vaginal epithelium or serious adverse events. Although this could have resulted in a bias, the main study question was to determine the efficacy of cleaning; this inherently applies to patients who are in fact successful users.

Second, comparison of the 3 months and 9 months cleaning intervals might be deceptive. It is possible that the measurement of vaginal symptoms after a 9-month interval is actually a reflection of a bacterial environment that had already colonized the vagina in the preceding 6 months. What we can conclude is that lengthening the cleaning interval does not increase discharge and therefore most probably does not lead to an increase in colonization [8–10].

Third, the inclusion of patients performing self-management may have influenced cleaning and interval results in this group of patients. However, we decided to offer education on self-management to all of our patients, as the ability for self-management is desirable; the self-reliance of patients increases and the need for a doctor’s visit diminishes. As a result, instruction for self-management is increasingly becoming part of normal routine. Therefore, we decided to include this group [20, 21].

Finally, there was a considerable amount of missing data in this study. Most of the missing data consisted of post-visit VAS measurements. Patients received the post-visit questionnaire and instructions at the respective follow-up visit. To simplify returning these documents, patients also received an addressed envelope. If the questionnaire was not returned after 2 weeks, it was noted as missing. At this time, new questionnaires were not sent because the results, when obtained too long after the specific visit, could no longer be related to the actual event of cleaning.

### Interpretation of results

The first question of this study was whether the actual cleaning of a pessary is effective in reducing symptoms. In this study, no significant differences in pain, discharge and irritation were found when the situation before and after cleaning was compared.

For this finding, there can be several reasons.

First, the highest median VAS scores in pessary users were found for vaginal discharge. This is consistent with other studies on this topic [4, 5, 7, 8, 10]. Still, in this study we only found a median VAS score of as low as 3 on a ten-point scale (range 1–5) for vaginal discharge. A relatively low pre-existing VAS score before cleaning can be partly responsible for the absence of a measurable effect of cleaning.

Second, the susceptibility for the development of vaginal discharge differs between individuals. Differences in vaginal environment and pre-existing acidity (presence of lactobacillus and atrophy) are known to influence the development of colonization and inflammation [8–10, 22].

Considering that the vagina itself is not cleaned during the cleaning visits, our hypothesis is that it is not likely that the short-term removal of the pessary, cleaning and replacement will substantially alter bacterial colonization, inflammation or vaginal acidity. This is underlined by the fact that we did not find differences in our population between the pre- and post-cleaning visit.

The second question of this study was whether the length of the interval between cleaning visits influences pessary-related side effects (vaginal pain, discharge and irritation). It was shown that an interval of 3 months versus a 9-month interval did not influence the severity of pessary-related side effects. In the literature, it is often recommended to apply intervals of between 3 to 6 months [1, 12, 16, 17]. However, we found no evidence to support these recommendations. The severity of side effects, particularly discharge (colonization of the vaginal microenvironment) appears to develop in the first period after insertion of a pessary and remains relatively stable over time [8–10]. Groups that studied the effect of pessaries on the microenvironment of the vagina showed that microscopic colonization changes accompanied by an increase in bothersome discharge already develops and occurs during the first 2 weeks of pessary use and reaches a steady state in the next 3 months [8]. Other studies have confirmed that changes in microenvironment occur mainly during the first period of pessary use [9, 10]. The finding in our study that the severity of discharge does not increase over time is therefore in line with the literature.

Having established that intervals do not matter for the most bothersome complaints, it still might be worthwhile to remove the pessary owing to a possible chance of lesions of the vaginal epithelium and subsequent epithelial overgrowth or fistula. Other studies showed that serious adverse events in women with pessary treatment were very rare and mostly due to longstanding neglect [4, 5, 12]. In our study, we did not find any serious adverse events in patients with continued pessary use after 12 months.

Finally, our third question was which proportion of patients was willing and able to remove, clean and re-insert their pessary.

Almost half of patients were able to learn and continue self-management after 12 months. Comparable percentages were described in a study by Kearney et al. They showed that 65% of patients were able to learn self-management and that 47% continued self-management after 6 months’ follow-up [23].

To implement the outcomes of our study into clinical practice and subsequently reduce health care costs, we recommend a routine follow-up visit not more frequently than at 9-month intervals in a symptomatic patient without self-management. We have not studied longer intervals and therefore we do not know whether serious complications would occur at larger intervals. In our opinion, there is no evidence for the need to check asymptomatic patients performing self-management. Individualized care and visits should be provided on the indication of complaints of increasing vaginal discharge (exclude or treat bacterial vaginosis), blood loss or (increasing) pain in all patients.

## Conclusion

Frequent pessary cleaning in asymptomatic women with POP is not efficacious with regard to pessary-related side effects such as pain, discharge and irritation. There is no difference in the severity of these pessary-related side effects between a 3- and a 9-monthly follow-up interval.

## Abbreviations

POP: Pelvic organ prolapse
VAS: Visual analogue scale
POP-Q: Pelvic organ prolapse quantification
IRB: Institutional review board
SUI: Stress urinary incontinence
CRF: Case record form
SPSS: Statistical Package for the Social Sciences
